# Case Series: Gene Expression Analysis in Canine Vogt-Koyanagi-Harada/Uveodermatologic Syndrome and Vitiligo Reveals Conserved Immunopathogenesis Pathways Between Dog and Human Autoimmune Pigmentary Disorders

**DOI:** 10.3389/fimmu.2020.590558

**Published:** 2020-12-15

**Authors:** Ista A. Egbeto, Colton J. Garelli, Cesar Piedra-Mora, Neil B. Wong, Clement N. David, Nicholas A. Robinson, Jillian M. Richmond

**Affiliations:** ^1^ Department of Dermatology, UMass Medical School, Worcester, MA, United States; ^2^ Tufts University School of Medicine, Boston, MA, United States; ^3^ Pathology Department, Tufts Cummings School of Veterinary Medicine, Grafton, MA, United States; ^4^ Nanostring Technologies, Seattle, WA, United States

**Keywords:** vitiligo, Vogt-Koyanagi-Harada disease, immunopathogenesis, transcriptomics, comparative immunology, canine (dog)

## Abstract

Vogt-Koyanagi-Harada syndrome (VKH) and vitiligo are autoimmune diseases that target melanocytes. VKH affects several organs such as the skin, hair follicle, eyes, ears, and meninges, whereas vitiligo is often limited to the skin and mucosa. Many studies have identified immune genes, pathways and cells that drive the pathogeneses of VKH and vitiligo, including interleukins, chemokines, cytotoxic T-cells, and other leukocytes. Here, we present case studies of 2 canines with VKH and 1 with vitiligo, which occurred spontaneously in client-owned companion dogs. We performed comparative transcriptomics and immunohistochemistry studies on lesional skin biopsies from these cases in order to determine if the immunopathogenesis of autoimmune responses against melanocytes are conserved. In dogs, we found enrichment of T cell gene signatures, with upregulation of IFNG, TNF, PRF1, IL15, CTSW, CXCL10, and CCL5 in both VKH and vitiligo in dogs compared to healthy controls. Similar findings were reported in humans, suggesting that these genes play a role in the pathogenesis of spontaneous VKH and vitiligo. T cell-associated genes, including FOXP3 and TBX21, were enriched, while IGFBP5, FOXO1, and PECAM1 were decreased compared to healthy controls. Further, we identified TGFB3, SFRP2, and CXCL7 as additional potential drivers of autoimmune pigmentary disorders. Future studies exploring the immunopathogenesis of spontaneous autoimmunity will expand our understanding of these disorders, and will be useful in developing targeted therapies, repurposing drugs for veterinary and human medicine, and predicting disease prognosis and treatment response.

## Introduction

Autoimmune pigmentary disorders include vitiligo and Vogt-Koyanagi-Harada (VKH) syndrome, which are caused by the destruction of melanocytes (1, 2). These diseases are mediated by T-cells that target melanocyte self-antigens, including tyrosinase, tyrosinase-related proteins 1 & 2 (TRP1/2), gp100/Pmel-17, and melan-a/MART-1 (3–9). Vitiligo is characterized by loss of melanocytes in the skin and mucosa (1). VKH is more extensive and includes the skin, mucosa, eyes, ears, and meninges, resulting in chronic uveitis, alopecia, vitiligo, poliosis, and irritation of the meninges (10). The current hypothesis in the field is that VKH represents an exacerbated reaction by melanocytes and their precursors as compared to vitiligo.

VKH is usually manifested during the third decade of life and is present in all ethnic groups across the world. The prevalence is higher in groups with darker skin tones and in Asians (11). VKH syndrome is thought to be associated with HLA-DRB1*0405 (12), and polymorphisms in immune genes (13). Patients often present with bilateral uveitis that is often preceded by a mild prodromal illness, along with auditory and neurological symptoms. Isolated ocular involvement in the initial phases of the disease is also common. The choroid is the primary site of inflammation, with the iris and ciliary body also affected. VKH syndrome is classified into three different categories:

1. Complete VKH- vitiligo is associated with complete VKH. Manifestations of complete VKH includes diffuse choroiditis affecting the eyes bilaterally and causing retinal detachments. Other signs include tinnitus, alopecia, neck stiffness, poliosis, and vitiligo.
2. Incomplete VKH- these patients present with similar ocular symptoms as patients with complete VKH, but they do not have both neurological and skin symptoms.
3. Probable VKH- these patients have similar ocular symptoms as patients with VKH, and they tend to have neurologic and auditory manifestations or dermatologic signs, but not both (10).

Several cytokines and chemokines contribute to the development of vitiligo and VKH, including type 1 responses (IFNγ, CXCL9/10/11, IL-12, TNF) type 17 responses (IL-17, CCL20, IL-23) and IL-2, which supports T cell growth and survival (13–19). Some studies have demonstrated that patients with VKH that have vitiligo have a predominance of CD4+ T-cell lymphocytes and an imbalanced ratio of CD4+/CD8+ T-cells (10), though others have demonstrated a CD8+ cytotoxic T cell preponderance (15). These CD8+ T cells react to melanocyte antigens and exhibit markers of skin resident memory T cells (20, 21).

Vitiligo and VKH in canines share similar clinical characteristics with human vitiligo and VKH [reviewed in (22)]. Dogs that have VKH present similarly to humans with incomplete VKH, exhibiting panuveitis and bilateral retinal detachment. Here, we analyzed lesional skin tissue from two dogs with VKH and one dog with vitiligo who presented to community veterinary clinics and were biopsied for diagnostic purposes. We performed transcriptomic and immunohistochemistry analysis on lesional skin tissue to examine features of immunopathogenesis of pigmentary disorders that are conserved during spontaneous disease in dogs versus humans. Our findings support the IFNγ-CXCR3 axis as a prominent feature in canine autoimmune pigmentary disorders. We also identified TGFB3, SFRP2 and CXCL7 as other potential drivers of immunopathogenesis.

## Case Presentations

### Case 1

A 2.5-year-old male Bernese Mountain dog that presented with a 6-month history of loss of pigmentation on his nose with periocular erythema, seborrhea, and crusts around eyes, on the nose, back, and tail. These symptoms were resolved with three months of steroid therapy. Two punch biopsies were performed, and histopathology revealed a lichenoid inflammation that multifocally obscured the dermo-epidermal junction (interface inflammation). The inflammation is predominantly composed of macrophages and fewer lymphocytes and plasma cells. There was significant pigmentary incontinence in the areas of inflammation with macrophages containing fine, dust-like, granular melanin pigment. The epidermis was moderately hyperplastic and hyperkeratotic. All the above findings were consistent with VKH-like syndrome.

### Case 2

A 4-year-old neutered male Siberian Husky dog that presented with a 2-month history of changes of pigmentation and pruritus of the nasal planum, muzzle, periocular skin, and oral mucous membranes. The dog developed blepharospasm, iridal color change, and pupillary miosis in the left eye. A punch biopsy in this dog revealed similar histopathological findings as in case 1 that were all consistent with VKH-like syndrome.

### Case 3

A 1.5-year-old, neutered male Rottweiler-Labrador mixed dog presented with 1-month history of pigment changes of the nose and haircoat. On physical examination the dog had bilaterally symmetrical areas of leukotrichia interspersed with pigmented (black) hair and no evidence of erythema, alopecia, or crusting. Histopathology patchy and sharply demarcated loss of melanocytes in the epidermis and follicular epithelium. Multifocally, the epidermis and follicular epithelium, particularly in samples from the planum nasale, there is a mild, perivascular lymphocytic infiltrate with frequent migration of lymphocytes into the epidermis. Some hair shafts contained minimal pigment. In light of the clinical and histopathological findings, a diagnosis of vitiligo was made.

## Results

### Gene Expression Analysis From Case Series Reveals Both New and Previously Identified Genes Relevant to Anti-Melanocyte Autoimmunity

**Figure 1:**
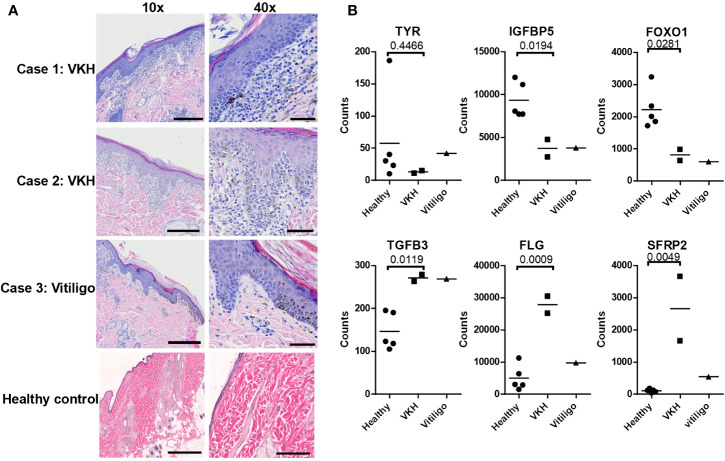
Examination of histopathology, skin, and hair gene expression patterns in VKH and vitiligo in dogs reveals marked infiltration with changes in specific genes. **(A)** Histopathologic examination of skin tissue samples. Hematoxylin and eosin (H&E) staining, magnifications 10x and 40x (scale bar = 300 microns 10x, scale bar = 60 microns 40x). There is marked inflammation at the dermo-epidermal junction (interface dermatitis) in VKH (cases 1 and 2) and epidermal hyperplasia in vitiligo (case 3), with pigment incontinence noted in all cases. **(B)** Relative mRNA expression of skin and hair-related gene transcripts in the cases compared to 5 healthy controls (leg margins). TGFB3, FLG, and SFRP2 were significantly increased, and TYR, IGFBP5, and FOXO1 were decreased; n = 2 VKH, 1 vitiligo, and 5 healthy controls; *p* values from two-tailed t tests healthy vs VKH: TYR p = 0.4466, IGFBP5 p = 0.0194, FOXO1 p = 0.0281, TGFB3 p = 0.0119, FLG p = 0.0009, SFRP2 p = 0.0049.

RNA was extracted from two VKH cases, one vitiligo case, and five healthy control leg margins and was analyzed with a custom Nanostring probeset (H&E from cases presented in **Figure 1A**). Agglomerative clustering of the entire 160 codeset revealed that the three pigmentary disorder cases grouped together apart from healthy controls (**Figure S1**). To understand the biology of VKH and vitiligo in dogs, we performed more detailed analyses on subsets of genes based on known cellular functions. First, we examined genes related to skin and hair biology, as well as neuroendocrine function in the skin (see **Figure S2** for complete gene set analyses). Tyrosinase (TYR) transcript was expressed in the healthy controls and the vitiligo case, while the mean trended lower in the VKH cases but showed no statistical difference from controls (**Figure 1B**).IGFBP5, which is known to mediate fibrosis (23), and FOXO1, which is associated with adipogenesis (24), were significantly downregulated in lesional skin (**Figure 1B**). These genes have not previously been associated with vitiligo or VKH, thus their functions in disease warrant further investigation. TGFB3, a growth factor and immune modulator, was significantly upregulated in both VKH and vitiligo (**Figure 1B**). Profilaggrin (gene name FLG), a protein hormone that promotes hair growth, and SFRP2, a soluble regulator of WNT signaling, were significantly upregulated in VKH but not vitiligo (**Figure 1B**). WNT signaling has previously been reported to be disrupted in lesional vitiligo skin (25), though its role in VKH is still unclear. Other skin-relevant transcripts including involucrin (IVL), LORICRIN, EDA, EDAR, KIT, WIF1, DNMT1, RXRG, VDR, TGFB1/2, and DCT showed no statistical differences from controls (**Figure S2**). The neuroendocrine gene CYP1B1 was significantly downregulated in cases versus controls, though this contrasts with a previous report describing upregulation in vitiligo blood samples (Dey-Rao and Sinha 2017). KRT10, a marker of basal keratinocytes, was significantly higher and PECAM1, a marker of endothelial cells, was significantly lower in cases versus healthy controls, which may reflect the site of biopsy (e.g. healthy leg margin versus nose for cases; **Figure S2**).

**Figure 2:**
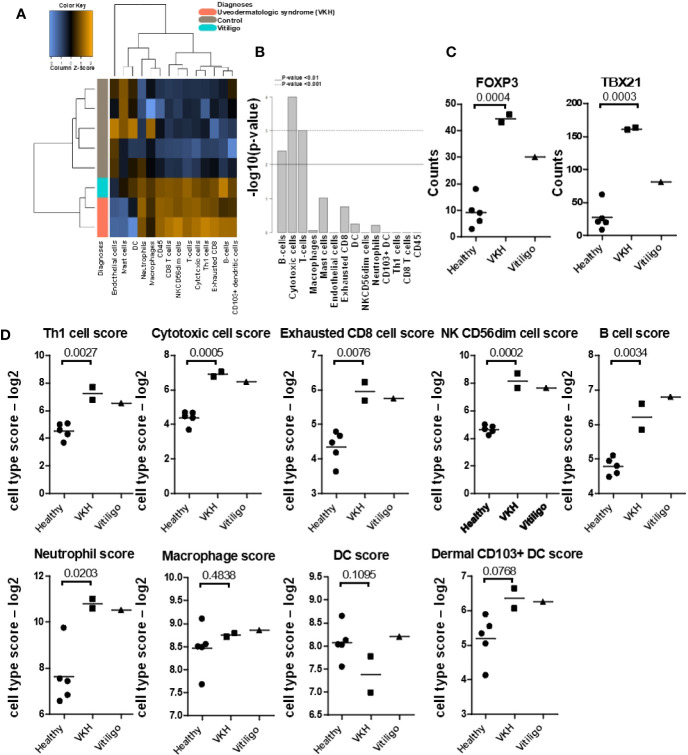
Quantification of cell types and mRNA expression of T-cell-associated transcription factors FOXP3 and TBX21/Tbet reveals cytotoxic and Th1 responses. **(A)** Nanostring advanced cell type analysis heat map representing color-coded expression levels of differentially expressed genes from the indicated leukocyte populations. Cases clustered together apart from healthy controls. **(B)** Bar plots of p-values for cell type enrichment analysis revealed that cytotoxic T-cells, B-cells, and cytokines are the predominant cells in VKH samples. **(C)** FOXP3 and TBX21 were significantly increased in VKH samples as compared to healthy controls; *p* values from two-tailed t tests FOXP3 p = 0.0004, TBX21 p = 0.0003. **(D)** Cell type scores from Nanostring advanced analysis demonstrating significant increases in Th1 cells, cytotoxic cells, exhausted CD8s, NK CD56dim cells, B cells, and neutrophils in VKH cases. Macrophage and DC scores remained unchanged, whereas dermal CD103+ dendritic cell scores were significantly increased as compared to healthy controls. n = 2 VKH, 1 vitiligo, and 5 healthy controls; *p* values from two-tailed t tests: Th1 p = 0.0027, Cytotoxic p = 0.0005, exhausted CD8 p = 0.0076, NK CD56dim p = 0.0002, B cell p = 0.0034, neutrophil p = 0.0203, macrophage p = 0.4838, DC p = 0.1095, dermal CD103+ DC p = 0.0768.

Next, we used Nanostring Advanced Analysis software to quantify cell types across our samples. We found that, similar to humans (5, 26), T-cells are the predominant immune cells found in VKH and vitiligo compared to healthy controls (**Figures 2A, B**). We examined the T-cell-associated transcription factors FOXP3 and TBX21 (Tbet), which are the master regulators in Tregs and Th1/Tc1 cells respectively, and found they were induced in VKH and vitiligo compared to controls (**Figure 2C**). Th1 cells, cytotoxic cells, exhausted CD8 T-cells and NK cells gene expression signatures were increased in VKH and vitiligo compared to healthy controls (**Figure 2D**). Neutrophil and B cell signatures were also increased in lesional skin, whereas macrophage and dendritic cells (DC) scores were equivalent across all 3 conditions. However, there was an increase in CD103+ dermal DC scores, which are known to cross-present antigens during antiviral and anti-tumor immune responses (27, 28). The full analysis of the canine immune cell panel genes is presented in **Figure S3**.

**Figure 3:**
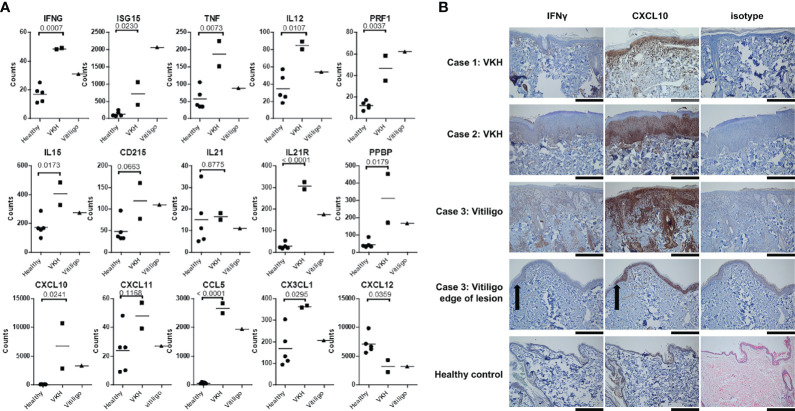
Many cytokines and chemokines previously associated with human VKH and vitiligo are enriched in canine lesional skin. **(A)** Relative mRNA expression of IFNG, ISG15, TNF, IL12, PRF1, IL15, IL21R, CXCL7, CXCL10, CCL5, and CX3CL1 were statistically significantly higher in VKH samples as compared to healthy controls. CXCL12 mRNA expression was significantly lower in VKH samples compared to healthy controls, CD215 and CXCL11 were trending higher (though they did not reach statistical significance), and IL21 was not significantly different. n = 2 VKH, 1 vitiligo, and 5 healthy controls; *p* values from two-tailed t tests healthy vs VKH: IFNG p = 0.0007, ISG15 p = 0.023, TNF p = 0.0073, IL12 p = 0.0107, PRF1 p = 0.0037, IL15 p = 0.0173, CD215 p = 0.0663, IL21 p = 0.8775, IL21R p < 0.0001, CXCL7 p = 0.0179, CXCL10 p = 0.0241, CXCL11 p = 0.1168, CCL5 p < 0.0001, CX3CL1 p = 0.0295, CXCL12 p = 0.0359. **(B)** Immunohistochemistry stains for IFN-γ and CXCL10 expression in lesional skin biopsies from dogs demonstrating increased protein levels in cases as compared to isotype and healthy controls (black scale bars = 300 microns 10x, grey scale bar = 150 microns 20x, black arrow case 3 indicates edge of lesion). n = 2 VKH, 1 vitiligo, and 5 healthy controls.

We next analyzed cytokines and chemokines. We found statistically significant induction of IFNG, ISG15, TNF, IL12, PRF1, and IL15 cytokine expression in dogs with VKH compared to healthy controls (**Figure 3A**). The vitiligo case exhibited even higher ISG15 and PRF1, while IFNG, TNF, IL12, and IL15 levels were closer to those in healthy controls. All of these genes have been reported to be induced in immune-mediated pigmentary disorders (21, 29–31). There was a trend towards increased CD215, a receptor chain for IL15 that has previously been shown to be upregulated on keratinocytes from lesional skin (21), in the cases. IL21, which has previously been reported to be upregulated in the Smyth chicken line, another model of spontaneous vitiligo (32), was unchanged in our samples. However, the IL21R was highly upregulated in lesional skin in the VKH and vitiligo dogs. The full analyses of the immune disease related genes, interferons and granzymes, and interleukins/cytokines are presented in **Figure S4**.

Mouse and human studies have shown that the chemokines CCL5 and CXCL10 are expressed in the skin during vitiligo (14, 15, 33, 34). We found significant upregulation of CCL5 and CXCL10, as well as CXCL7 (gene name PPBP) in canine VKH and vitiligo. CX3CL1 was significantly upregulated in VKH only, and there was a similar, though not significant, trend in CXCL11 upregulation. CCL2 (**Figure S3**) and CXCL12 (**Figure 3A**) were significantly downregulated in both conditions compared to healthy controls. The full analysis of chemokine expression is presented in **Figure S5**.

### IFNγ and CXCL10 Protein Expression Are Increased in Lesional Skin of VKH and Vitiligo Compared to Healthy Controls

To confirm that IFNG and CXCL10, two well-characterized drivers of anti-melanocyte responses, are expressed at the protein level in lesional skin of VKH and vitiligo canines, we performed immunohistochemistry. These cytokines were highly expressed in tissue as compared to isotype and healthy controls, with higher CXCL10 expression consistent with its role in amplifying IFN**γ** signals (**Figure 3B**). Taken together, these results characterize immune and skin gene expression in canine VKH and vitiligo, identify novel potential drivers of disease, and reveal conserved immunopathogeneses between human and canine spontaneous disease.

## Discussion

The pathogeneses of vitiligo and VKH are complex and not fully defined. To better understand driving factors of the autoimmune response in spontaneous autoimmune pigmentary disorders, we performed this retrospective comparative immunology case study to examine transcriptomics and immunohistochemistry of many known, and several unknown, genes central to vitiligo and VKH pathogeneses. In our study, we found increased T cell responses, type 1 cytokines, chemokines, and memory T cell responses in VKH dogs compared to healthy controls. These genes have been established as drivers of human vitiligo immunopathogenesis, particularly the IFNG cytokine signaling pathway (29, 35, 36) and subsequent CXCR3 ligand expression following activation of Janus Kinases (JAK) 1 and 2 (14, 37–40). CCR5 and CXCR3 expressing leukocytes are recruited to the skin following their ligands CCL5 and CXCL10, respectively (15, 33, 39). IL15 promotes development of skin resident memory T cells (41), and a recent studies in a vitiligo mouse model and human tissues revealed that the IL15 receptor is important for autoimmune memory in vitiligo (21). Other groups have reported that IL10, IL13, IL17A, and IL21 are increased in vitiligo (42–45), though we did not observe this in our case series study. Further, it is unclear if these cytokine profiles are pertinent to specific clinical subtypes of disease, such as inflammatory vitiligo, or if they are upregulated at specific phases of disease or as a result of concurrent autoimmunity or infections.

Like vitiligo, VKH appears to be driven by type 1 T cell responses (46, 47), though eye involvement reveals Th17 signatures (48). CX3CL1 was upregulated specifically in VKH skin in our case studies. Fractalkine has only been explored in ocular disease, warranting further investigation in cutaneous VKH, especially to understand differences in the biology versus vitiligo (49).

We examined skin-specific genes that are associated with skin and hair biology, including TYR, IGFBP5, FOXO1, FLG, WIF1, and TGFB3. Of these genes, TGFB3 and FLG were found to be significantly upregulated, whereas IGFBP5, FOXO1 and WIF1 were downregulated. In contrast to our findings of increased TGFB3 ligand, polymorphisms in its receptor were not found to be associated with VKH disease in a Chinese Han population (50). The disparity in TGFB3 expression could be explained by differences in dog versus human VKH, the stage at which the skin biopsies have been studied, and/or differences in the biology of the ligand versus the receptor. For example, TGFB3 (along with IL10) is expressed in the resolution phase of VKH and may play a relevant role in controlling the disease (51). The upregulation of FLG in VKH and not in vitiligo was an unexpected finding. We hypothesize that increased FLG expression in VKH dogs suggests a process of chronic inflammation in hair follicles, which is lacking in vitiligo. These differences may also be due to dog versus human pathogenesis, or perhaps due to disease stage/duration. We suggest further studies are needed to determine the roles of TGFB3 and FLG in VKH and vitiligo.

IGFBP5, FOXO1, and WIF1 downregulation may indicate a loss of tolerance mechanisms or melanocyte regenerative capacity in the skin in canine VKH and vitiligo. IGFBP5 acts as a tumor suppressor in human melanoma cells (52), which is interesting given the hypothesis that vitiligo exists on an “immune spectrum” with melanoma (53). FOXO1 represses TBX21-mediated effector functions to promote memory CD8+ T cell formation and Treg function; thus, a loss of FOXO1 may drive continued T cell effector function in the skin during melanocyte autoimmunity (54–56). WIF1 promotes melanogenesis in normal human melanocytes (57), though it is not yet clear what impact a loss of expression would have on vitiligo or VKH.

We also investigated genes involved in T-cell and Treg regulation and function: PPARG, FOXO3A, TBX21, and FOXP3. PPARG, which activates growth of melanocytes through apoptosis (58) and promotes T cell differentiation and survival (59, 60), was decreased in our cases compared to healthy controls. FOXO3 is a transcription factor that is an important regulator of the magnitude of CD8 T cell memory (61). FOXO3A polymorphisms have been associated with oxidative stress and altered Treg function in vitiligo patients (62, 63). Thus, decreases in PPARG and FOXO3 may indicate reduced ability of Tregs to function to suppress autoimmune responses of T cells against melanocytes. FOXP3 and TBX21 were increased in our cases compared to healthy controls, which correlates with data revealing increased expression of these transcription factors in VKH patients during an active uveitis episode (64). Together, our data support enhanced effector T cell populations and reduced Treg function present in the skin of canine vitiligo and VKH patients.

TYR, which we hypothesized would be significantly downregulated in VKH due to melanocyte loss, was not statistically different from the healthy controls. Notably, a study investigating tyrosinase gene family loci in VKH in Japanese patients using single microsatellite marker analysis and haplotype analysis did not find an association between TYR loci and VKH syndrome (65). This suggests that perhaps immature melanocytes or melanocyte precursors are preferentially targeted in VKH. Vitiligo was found to be associated with the major alleles of SNPs in the *TYR* region, particularly rs1393350 and the R402Q SNP rs1126809 (66). This may suggest a difference in pathogenesis between vitiligo and VKH involving tyrosinase as an autoantigen.

Our chemokine analysis revealed indicators of active, though not very early, disease in the dogs as evidenced by high CCL5 and FOXP3 (transiently upregulated in activated T-cells, allowing for the development of peripheral/induced regulatory T-cells) and low CXCL12 (67). We also identified PPBP/CXCL7 as a significantly upregulated chemokine in the skin. CXCL7 was previously reported to be upregulated in the serum of vitiligo patients, though little is known about its role in skin homing, warranting further study (68).

Our immune cell signature findings are strongly supported by previous studies that vitiligo and VKH are mediated by T-cells, particularly CD8 cytotoxic T-cells (69). Neutrophils are understudied in the skin in vitiligo and VKH, in part because they comprise a small fraction of the infiltrate and are not a prominent component. The increase in neutrophils we observed in these cases may be due to scratching behavior in the dogs, which is known to induce neutrophil recruitment to the skin (70). Nevertheless, blood neutrophils can contribute to ROS generation (71) and were reported to be elevated in the peripheral blood of patients with generalized vitiligo (72). We also observed increased B-cell signatures. It has been reported that in Akita dogs with VKH-like syndrome, cutaneous lesions are mediated by T cells and macrophages and ocular lesions are mediated primarily by B cells and macrophages (73). Autoantibodies against melanocytes have been identified in vitiligo and VKH, though it is still unclear if they are biomarkers or drive pathogenesis (74, 75). In lupus, another autoimmune disease, B cells have been postulated to serve as antigen presenting cells (76). Future studies will need to be conducted to determine the precise role of B cells and neutrophils in vitiligo and VKH.

In contrast to increases in other immune cell signatures, we found similar levels of macrophage and dendritic cell gene signatures in lesional skin tissue. Previous studies have noted the presence of macrophages in vitiligo lesions (6); thus a limitation of our data is that we did not examine localization of these cells within the tissue. It could be that, while total numbers of antigen presenting cell populations remain largely unchanged, that their localization within the tissue promotes lymphoid aggregates.

Limitations in our study include the small sample of genes analyzed (160 genes), and small sample size (we only had one vitiligo dog sample, thus preventing us from including the vitiligo sample in our statistical analyses). Due to the small group size of our VKH sample data, the t tests we performed may exhibit type I or type II errors which are dependent upon the variance of gene expression data within the groups (77). Nevertheless, several genes that we identified as differentially expressed between healthy and VKH have been previously published in human literature as DEGs for the condition. Future studies would include a larger scale comparative analyses and whole genome sequencing to better understand the influence of genetic factors on the pathogenesis of VKH disease and vitiligo.

In summary, our data support the hypothesis that T cell responses, type 1 cytokines, memory T cell responses, and chemokines drive immunopathogenesis of spontaneous VKH and vitiligo in both dogs and humans. Future studies expanding our understanding of spontaneous autoimmunity will be useful for providing a better understanding of autoimmune diseases and will pave the way for drug repurposing in human and veterinary medicine. For example, JAK inhibitors induce re-pigmentation in patients with vitiligo (78–81). Oclacitinib, a veterinary JAK inhibitor currently marketed for allergy and itch relief (82–85), could potentially be repurposed to treat vitiligo and VKH in dogs. Drugs that induce or worsen vitiligo could serve as a novel therapeutic approach to melanoma. A canine case report found a link between skin depigmentation and toceranib phosphate, a tyrosine kinase inhibitor (86), and the Flk-1 tyrosine kinase inhibitor SU5416 showed efficacy in phase II clinical trials for advanced melanoma (87). Future studies examining the mechanistic involvement of the gene targets we have identified in our case series for treatment of autoimmune pigmentary disorders or melanoma are warranted.

## Materials and Methods

### Clinical Samples

Skin biopsies from the biorepository at Tufts Cummings School (NR) were selected based on pathology reports and H&E sections were reexamined by a board-certified veterinary pathologist to confirm diagnoses and absence of obvious infectious disease. Healthy control samples were obtained from leg margin biopsies from amputations. Two VKH and one vitiligo biopsy sample were obtained from shave and/or punch biopsies of male dogs as noted in the case presentation section. Of note, vitiligo has equal sex bias in dogs, whereas VKH is almost twice as likely to occur in male dogs (22). Samples were deposited with written owner consent in the Tufts biobank at the time the veterinary patients were seen at the hospital, spanning the years 2011–2019.

### Isolation of RNA From FFPE Blocks

30 µm curls were cut from the blocks and stored in Eppendorf tubes at ambient temperature. RNA was isolated using the Qiagen FFPE RNeasy kit per the manufacturer directions. Briefly, razor blades were treated with RNase, excess paraffin was removed, and tissues were sliced into thin strips (5 μm) to create more surface area prior to incubation with deparaffinization solution (Qiagen). The protocol was followed and RNA was quantified using a nanodrop.

### Nanostring Cartridge and Processing

A custom Nanostring canine gene panel of 160 genes including cytokine, chemokine, and immune genes, as well as skin and immune cell specific transcripts was created. We used B2M, RPL13A, CCZ1, and HPRT as housekeeping genes for this study. RNA was hybridized using a BioRad C1000 touch machine, and samples were loaded into Nanostring cartridges and analyzed with a Sprint machine. Gene expression data are deposited on GEO under Accession # GSE154024.

### nSolver Analysis

NanoString’s software, nSolver was used for all NanoString analysis. Raw counts were plotted with GraphPad Prism. Advanced analysis was used for the “cell Type Score”, which is a summary statistic of the expression of the marker genes for each cell type. It is the geometric mean of the log2-transformed normalized counts for each set of marker genes. These scores were validated against FACS and IHC, and are a robust method of quantifying relative cell type abundance (88).

### IHC

IHC was performed on 5 µm sections using rabbit-anti-canine CXCL10, IFNγ (US Biological) or isotype control (Biolegend) at 1:100 dilution using a Dako automated slide staining machine. All sections were counterstained with hematoxylin. H&E images were taken using an Olympus BX51 microscope with Nikon NIS Elements software version 3.10, and IHC images were taken using an Olympus BX40 microscope with cellSens Entry software version 1.14.

### Statistics

To assess the statistical significance of our results, we performed two-way ANOVA with Bonferroni posttests between healthy controls and VKH cases on sets of defined genes (skin & hair, neuroendocrine, immune disease related, interleukins/cytokines, CC chemokines, CXC chemokines, immune cell panel genes, and housekeeping genes) to observe obvious differences across the dataset. We next performed two-tailed t-tests of VKH vs healthy using GraphPad Prism software version 5 to examine potential differences in previously identified genes pertinent to VKH pathogenesis (De Winter 2013). A statistically significant difference was considered as *p <*0.05.

## Data Availability Statement

The datasets presented in this study can be found on Gene Expression Omnibus (GEO) Database under Accession # GSE154024.

## Ethics Statement

The animal study was reviewed and approved by Cummings School of Veterinary Medicine at Tufts University IACUC. Written informed consent was obtained from the owners for the participation of their animals in this study.

## Author Contributions

Conceptualization: JMR. Methodology: NAR, JMR. Software: CND, CJG. Validation: CP-M, JMR. Formal analysis: IAE, NBW, JMR. Investigation: CJG, CP-M, NAR, JMR, CND. Resources: NAR, JMR. Data curation: NAR, CP-M, CJG, JMR. Writing—original draft: IAE. Writing—review and editing: all authors. Visualization: IAE, CP-M, NBW, JMR. Supervision: JMR, NAR. Project administration: JMR. Funding acquisition: JMR. All authors contributed to the article and approved the submitted version.

## Funding

JMR is supported by a Calder Research Scholar Award in Vitiligo/Pigment Cell Disorders from the American Skin Association, a Women's Health Career Development Award from the Dermatology Foundation, and a Target Identification in Lupus Award from the Lupus Research Alliance.

## Conflict of Interest

JMR is an inventor on patent application #15/851,651, “Anti-human CXCR3 antibodies for the Treatment of Vitiligo” which covers targeting CXCR3 for the treatment of vitiligo; and on patent #62489191, “Diagnosis and Treatment of Vitiligo” which covers targeting IL-15 and Trm for the treatment of vitiligo. CND is an employee of Nanostring Technologies.

The remaining authors declare that the research was conducted in the absence of any commercial or financial relationships that could be construed as a potential conflict of interest.

## Supplementary Material

Supplementary Figure 1 Heat map showing the expression patterns of the 160 gene Nanostring panel in cases and healthy controls. Purple represents upregulation and orange and yellow represent downregulation of gene expression. Color density is associated with the level of gene expression. Dendrogram indicates clustering/relatedness of samples, which segregated based on disease status.

Supplementary Figure 2 Expression of skin, hair, and neuroendocrine genes in VKH and vitiligo cases compared to healthy controls. The relative expression of **(A)** highly expressed skin and hair genes, **(B)** lower level transcript genes, and **(C)** neuroendocrine genes was evaluated by NanoString’s nSolver. n = 2 VKH, 1 vitiligo, and 5 healthy controls; *p* values from two-way ANOVA with Bonferroni post tests for healthy vs VKH: KRT10 p < 0.001, PECAM1 p < 0.001, SFRP2 p < 0.01, IGFBP5 p < 0.001, CYP1B1 p < 0.001.

Supplementary Figure 3 Expression of immune disease related genes in the autoimmune pigmentary cases versus healthy controls. **(A)** Immune related genes and **(B)** Interferons and granzymes expressed in skin. n = 2 VKH, 1 vitiligo, and 5 healthy controls; *p* values from two-way ANOVA with Bonferroni post tests for healthy vs VKH: SERPINB2 p < 0.05, VGLL3 p < 0.01, FAS p < 0.001, CTSW p < 0.01.

Supplementary Figure 4 Expression of interleukins/cytokines and chemokines in cases versus healthy controls. **(A)** Interleukins/cytokines, **(B)** CC chemokines, and **(C)** CXC chemokines in skin biopsies of VKH, vitiligo, and healthy samples. n = 2 VKH, 1 vitiligo, and 5 healthy controls; *p* values from two-way ANOVA with Bonferroni post tests for healthy vs VKH: IL6 p < 0.001, CCL2 p < 0.01, CCL5 p < 0.01, CXCL10 p < 0.01, CXCL14 p < 0.001.

Supplementary Figure 5 Expression of canine immune cell genes and housekeeping genes in cases versus healthy controls. **(A)** Highly expressed and **(B)** lower expressed canine cell panel genes. **(C)** Housekeeping genes in biopsies from cases and healthy controls. n = 2 VKH, 1 vitiligo, and 5 healthy controls; *p* values from two-way ANOVA with Bonferroni post tests for healthy vs VKH: PECAM1 p < 0.01, S100A12 p < 0.001, BLK p < 0.01, CD244 p < 0.001, CD6 p < 0.001, CD84 p < 0.001, GZMA p < 0.001, GZMB p < 0.001, IL21R p < 0.001, KLRD1 p < 0.001, KLRK1 p < 0.001, SH2D1A p < 0.001, TBX21 p < 0.001, TNFRSF17 p < 0.01, B2M p < 0.001.
