# Supplementary figures and images for: Case Series: Gene Expression Analysis in Canine Vogt-Koyanagi-Harada/Uveodermatologic Syndrome and Vitiligo Reveals Conserved Immunopathogenesis Pathways Between Dog and Human Autoimmune Pigmentary Disorders
Source: Front Immunol. 2020 Dec 15;11:590558. doi: 10.3389/fimmu.2020.590558 (PMC7770226; doi:10.3389/fimmu.2020.590558)

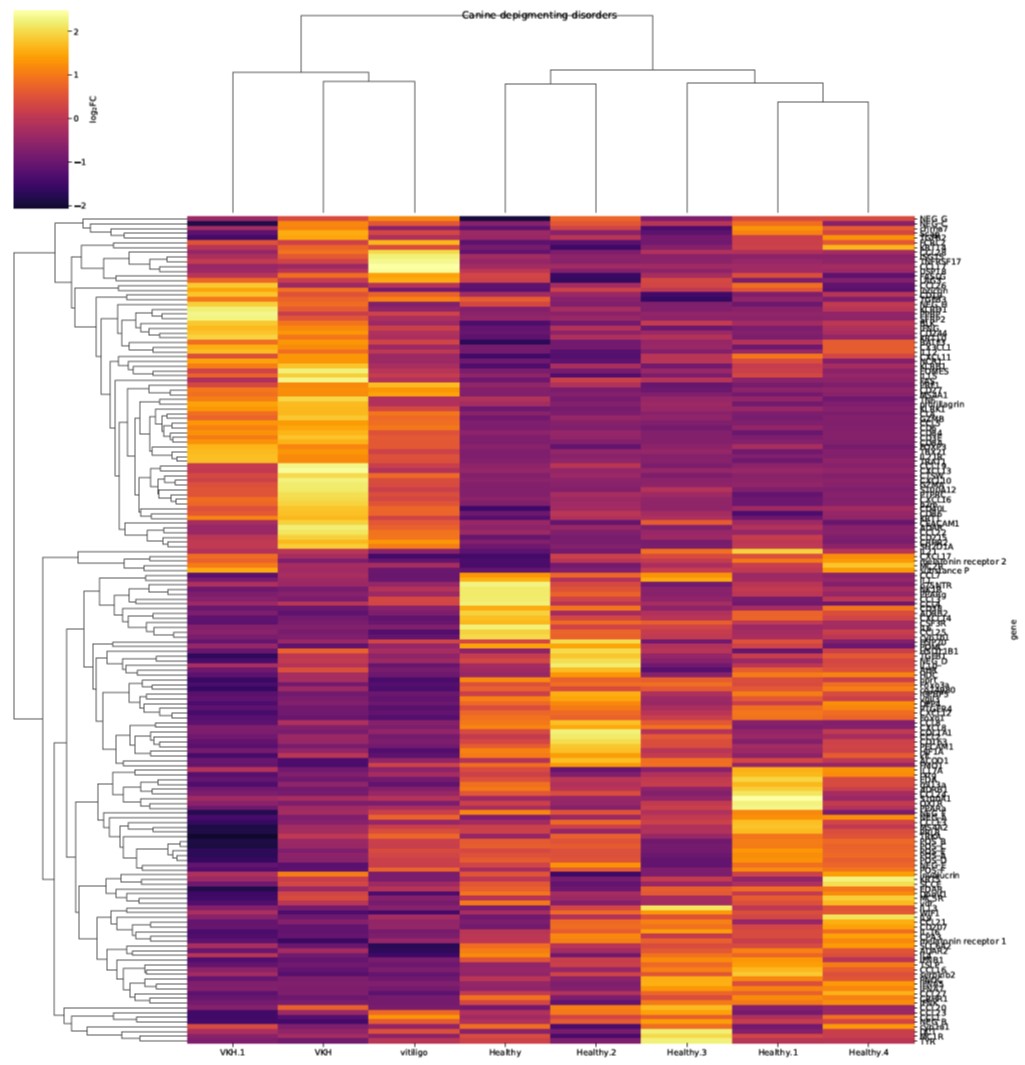

Supplement: Supplementary Figure 1 — Heat map showing the expression patterns of the 160 gene Nanostring panel in cases and healthy controls. Purple represents upregulation and orange and yellow represent downregulation of gene expression. Color density is associated with the level of gene expression. Dendrogram indicates clustering/relatedness of samples, which segregated based on disease status. [file Image_1.jpeg]

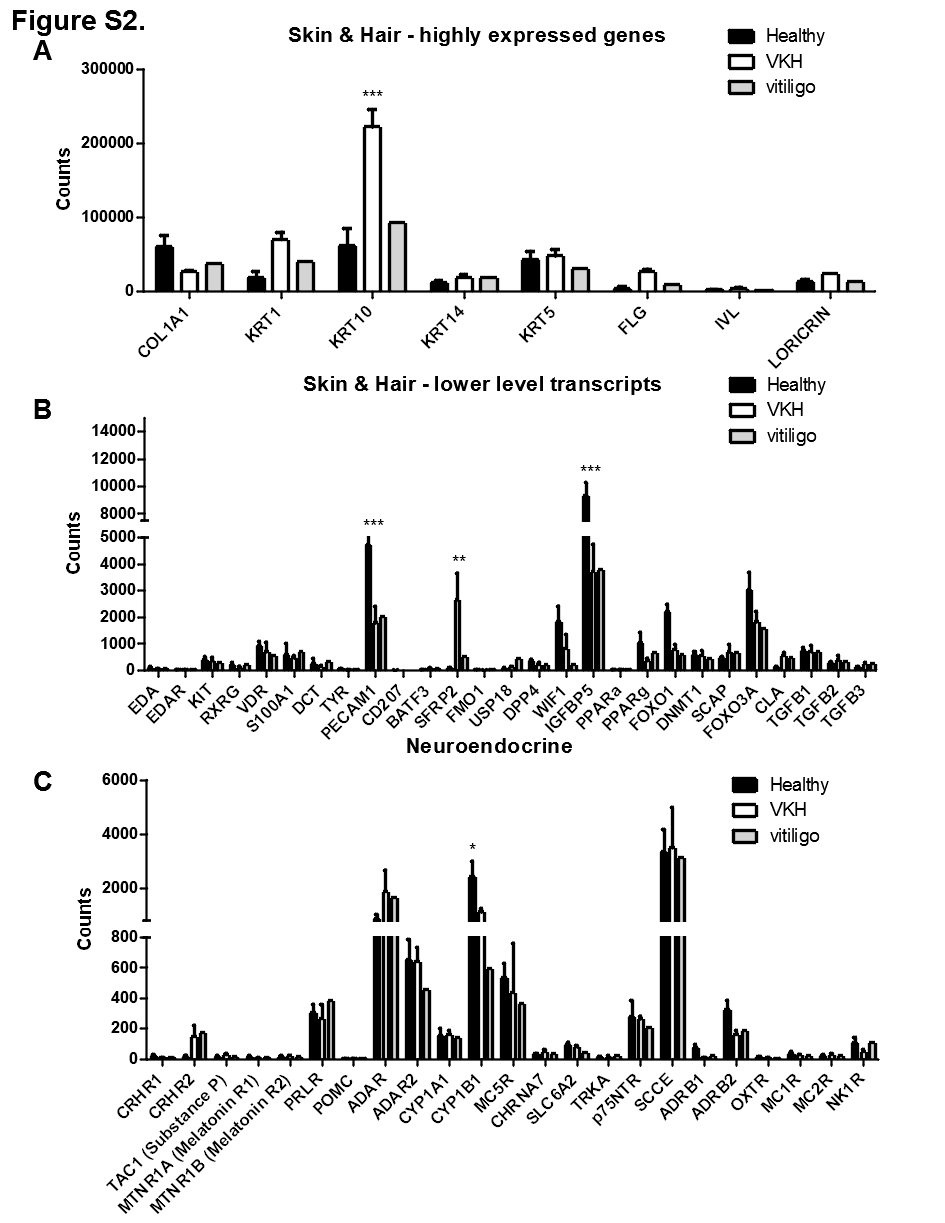

Supplement: Supplementary Figure 2 — Expression of skin, hair, and neuroendocrine genes in VKH and vitiligo cases compared to healthy controls. The relative expression of (A) highly expressed skin and hair genes, (B) lower level transcript genes, and (C) neuroendocrine genes was evaluated by NanoString’s nSolver. n = 2 VKH, 1 vitiligo, and 5 healthy controls; p values from two-way ANOVA with Bonferroni post tests for healthy vs VKH: KRT10 p < 0.001, PECAM1 p < 0.001, SFRP2 p < 0.01, IGFBP5 p < 0.001, CYP1B1 p < 0.001. [file Image_2.jpeg]

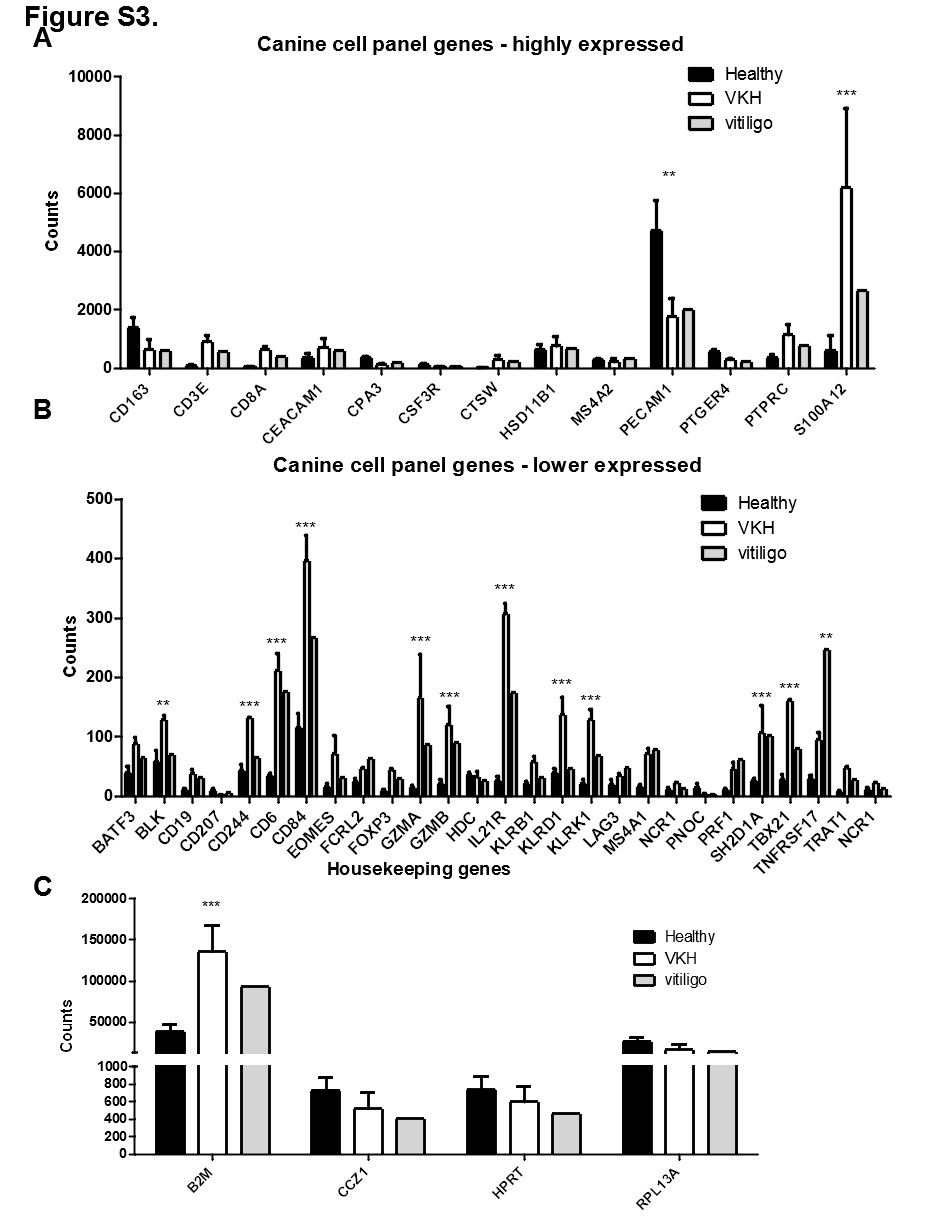

Supplement: Supplementary Figure 3 — Expression of immune disease related genes in the autoimmune pigmentary cases versus healthy controls. (A) Immune related genes and (B) Interferons and granzymes expressed in skin. n = 2 VKH, 1 vitiligo, and 5 healthy controls; p values from two-way ANOVA with Bonferroni post tests for healthy vs VKH: SERPINB2 p < 0.05, VGLL3 p < 0.01, FAS p < 0.001, CTSW p < 0.01. [file Image_3.jpeg]

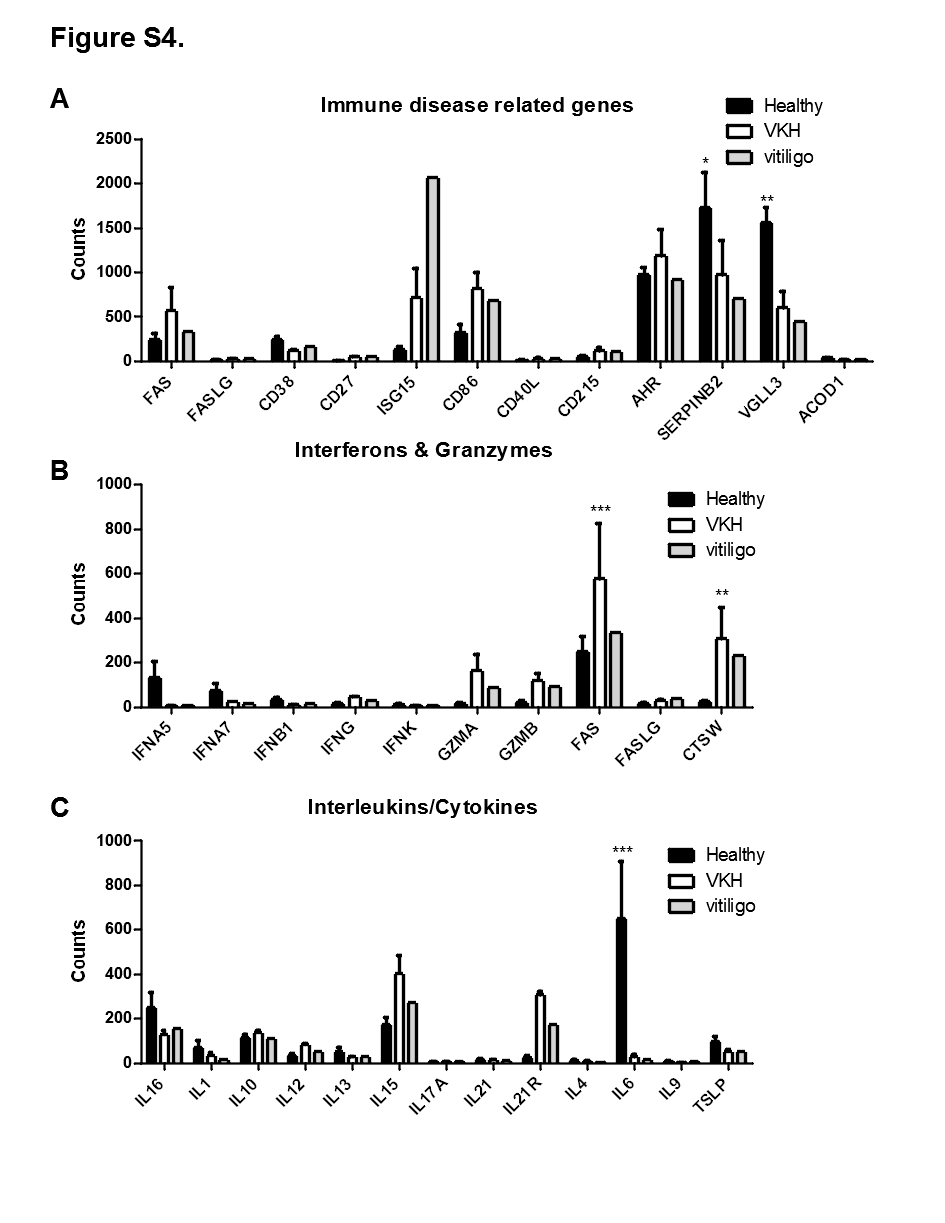

Supplement: Supplementary Figure 4 — Expression of interleukins/cytokines and chemokines in cases versus healthy controls. (A) Interleukins/cytokines, (B) CC chemokines, and (C) CXC chemokines in skin biopsies of VKH, vitiligo, and healthy samples. n = 2 VKH, 1 vitiligo, and 5 healthy controls; p values from two-way ANOVA with Bonferroni post tests for healthy vs VKH: IL6 p < 0.001, CCL2 p < 0.01, CCL5 p < 0.01, CXCL10 p < 0.01, CXCL14 p < 0.001. [file Image_4.jpeg]

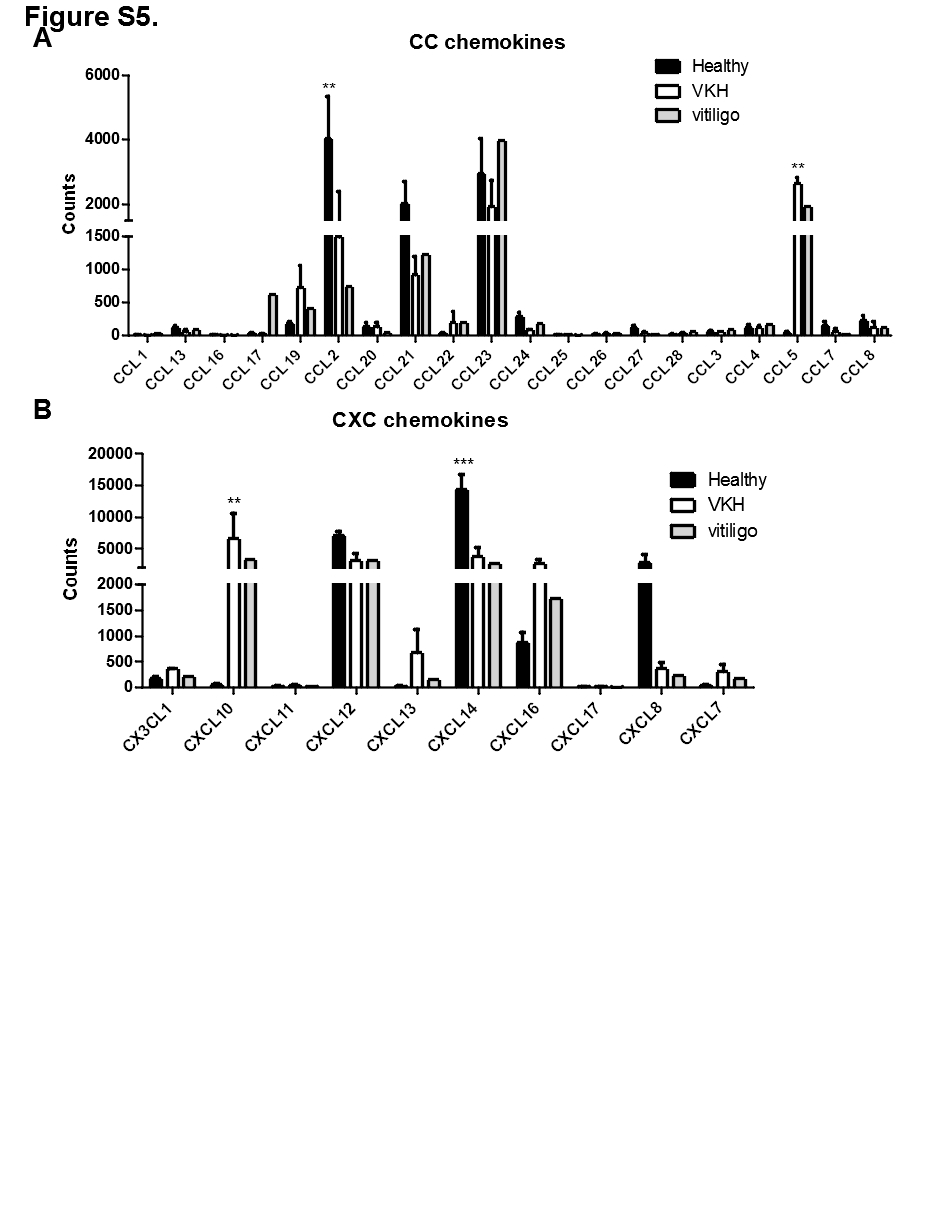

Supplement: Supplementary Figure 5 — Expression of canine immune cell genes and housekeeping genes in cases versus healthy controls. (A) Highly expressed and (B) lower expressed canine cell panel genes. (C) Housekeeping genes in biopsies from cases and healthy controls. n = 2 VKH, 1 vitiligo, and 5 healthy controls; p values from two-way ANOVA with Bonferroni post tests for healthy vs VKH: PECAM1 p < 0.01, S100A12 p < 0.001, BLK p < 0.01, CD244 p < 0.001, CD6 p < 0.001, CD84 p < 0.001, GZMA p < 0.001, GZMB p < 0.001, IL21R p < 0.001, KLRD1 p < 0.001, KLRK1 p < 0.001, SH2D1A p < 0.001, TBX21 p < 0.001, TNFRSF17 p < 0.01, B2M p < 0.001. [file Image_5.jpeg]
